# Examination of clinical conditions and chest CT images of Covid-19 cases in Turkey; single center study

**DOI:** 10.4314/ahs.v21i4.8

**Published:** 2021-12

**Authors:** Metin Ocak, Nur Şimşek Yurt, Yusuf Can Yurt

**Affiliations:** 1 Gazı State Hospital, Emergency Clinic Samsun, Turkey; 2 Samsun Training And Research Hospital, Family Medicine Clinic

**Keywords:** COVID-19, SARS-CoV-2, Chest CT, Ground Glass Opacity, Dyspnea, Cough

## Abstract

**Background:**

In this study, we aim to study the clinical features and chest CT findings of the patients, COVID-19 diagnosis of which are verified.

**Methods:**

This retrospective study was conducted on RT-PCR (+) COVID-19 patients who were examined in our hospital's emergency department between March 11, 2020 and June 30, 2020.

**Results:**

326 patients were included in this retrospective study in total. Of the patients, 53.1% and 46.9% are males and females, respectively. The patients applied to the service with the symptoms of shortness of breath at the rate of 21.8% and cough at the rate of 20.6%. The study found that the most frequent abnormal CT finding is ground glass opacity at the rate of 26.7% and it is followed by atelectasis at the rate of 12.3%. Patients in the> 65 age group and patients with COPD comorbidities have significantly higher rates of admission to the intensive care unit.

**Conclusions:**

The most frequent findings in examination of pathological chest CT findings are ground glass opacity. The rate of length of intensive care unit stay and mortality is significantly higher in the patients above 65 years old compared to those below 65 years old.

## Background

Coronavirus disease 2019 (COVID-19), a highly infectious disease caused by severe acute respiratory syndrome coronavirus 2 (SARS-CoV-2), was firstly reported in Wuhan, Hubei Province, China, and has rapidly spread to other domestic cities and countries beyond China 1,2. On January 30, 2020, the World Health Organization (WHO) declared this ongoing outbreak as a global public health emergency and raised the risk of COVID-19 to very high at the global level on February 28, 2020 3. WHO declared COVID-19 as a pandemic on March 11, 2020 4. SARS-CoV-2 is thought to have zoonotic origin and its place of origin is traced back to the wild animals sold in the marine products wholesale market. Symptomatic/asymptomatic people with COVID-19 positive are the infection origins of COVID-19 due to its capability of spreading from human to human. The disease basically spreads through droplets exhaled from breath 5. The first COVID-19 case in Turkey was recorded on March 11, 2020 considerably later than the neighboring countries such as European countries and Iran thanks to the early precautions taken across the country 6.

America and Europe continents; the highest numbers of cases were reported in USA, Brazil, Russia, India, and United Kingdom 7. The number of cases is soaring up all around the world; the number of confirmed COVID-19 cases worldwide as of March 25, 2021 is 123.942.242 and total number of deaths is 2.727.837. The number of cases and deaths in Turkey were reported 3.061.520 and 30.316 respectively 8.

Clinical spectrum of COVID-19 varies from asymptomatic, mild or moderate respiratory tract infections to serious cases that develop Acute Respiratory Distress Syndrome (ARDS) or multiple organ dysfunctions resulting in death. Therefore, early diagnosis and treatment of both asymptomatic and symptomatic cases have great importance in management of patients and reducing the spread of the disease to other members of the society 9. Reverse transcription-polymerase chain reaction (RT-PCR) and chest computer tomography (CT) are the main diagnostic methods of COVID-19. However, there is currently no consensus about which method is superior to the other. RT-PCR tests are known to provide considerable amount of false negative results. This is due to many reasons including inappropriate clinical sampling, different viral load levels, technical problems, and standardization difference among different laboratories and hospitals 10. Chest CT is recommended as an alternative and reliable method in diagnosis of COVID-19 patients in China 11. In literature, a number of studies are reported on the features of chest CT images of COVID-19 patients 9, 11,12–19.

In this study, we aim to analyze the clinical and demographic data and chest CT features of the verified COVID-19 patients tested positive in RT-PCR. We think that this study may contribute to the literature in radiologic evaluation and follow-up of COVID-19 patients.

## Methods

This retrospective study was carried out on 326 COVID-19 patients that are tested positive in RT-PCR and examined in Emergency Service of Samsun Gazi State Hospital.

Age, symptoms, vital findings, comorbid diseases, smoking habit, and pre-application 14-day clinical history of the patients were investigated in triage area of the emergency service and the patients diagnosed as potential or confirmed COVID-19 cases were directed to the isolation section, a separate area inside the service. Potential case is defined with at least one of the symptoms and findings of fever or respiratory tract disease (cough and shortness of breath), failure to explain the clinical indicators with other diseases, the fact that the patient or one of his/her relatives went abroad in the 14 days before the beginning of symptoms or the cases who were in close contact with a COVID-19 case. Confirmed cases are defined as the cases that answer to the description of potential case and are diagnosed with SARS-COV-2 RT-PCR tests from nasopharyngeal swab samples 5. The laboratory and chest imaging tests of the patients in the isolation section were carried out and the nasopharyngeal swab samples were taken from the people in close contact with a COVID-19 case for SARS CoV-2 RT-PCR test. Unenhanced chest CT scan was performed for chest imaging. Definition, location, and spread of lesions were observed in the chest CT images of the patients. Besides, the differences of pathological CT findings in the patients below and above 65 years old as well as in the recovered and dead patients were investigated.

### Data collection

Epidemiological, demographic, clinical, laboratory, treatment, and result data and chest CT images of the patients that were examined in the isolation section and diagnosed with RT-PCR (+) were obtained retrospectively from the patient files and electronic database of the hospital. The chest CT images of the patients were examined together with a radiology specialist.

### Ethics committee approval

The necessary ethical permission for the study has been obtained from the local ethics committee.

### Statistical Analysis

Descriptive statistics are given together with mean, standard deviation values for continuous data, and in numbers and percentages for categorical data. The rates of categorical variables were compared by using Chisquare test. Post-hoc analysis was performed to determine the group of significance. Bonferroni correction was applied in post-hoc analysis. Logistic regression analysis was performed to determine the predictive effect of age and chronic obstructive pulmonary disease (COPD) on admission to the intensive care unit. The p values at the confidence interval of 95% and below 0,05 were considered significant for statistical significance. The program IBM SPSS (Statistical Package for the Social Sciences, Chicago, IL, USA), version 21.0 was used for the statistical analysis.

## Results

326 patients were included in this retrospective study in total. Of the patients, 53.1% are males and 46.9% are females. While 1.5% of the patients are below 18 years old, 16.9% are above 65 years old. Mean age of the patients was found 42.39 ± 18.596 (14–92). The study found that the most common symptoms in the patients are shortness of breath (21.8%) and cough (20.6%). Demographic features of the participants and their symptoms during application are given in Table 1.

**Table 1:** Distribution of patients by certain socio-demographic features and symptoms

	PCR+ Cases	
Features	Number (N)	Percentage (%)
**Sex**		
Male	173	53.1
Female	153	46.9
**Age groups**		
18 years old and below	5	1.5
19–30 years old	116	35.6
31–50 years old	107	32.8
51–64 years old	43	13.2
65 years old and above	55	16.9
**Symptoms**		
Asymptomatic	141	43.3
Sore throat	15	4.6
Myalgia	29	8.9
Fatigue	8	2.5
Cough	67	20.6
Fever	23	7.1
Chest Pain	7	2.1
Shortness of Pain	71	21.8
Diarrhea/Nausea-vomiting	5	1.5
Headache/Dizziness	5	1.5
**Total**	**326**	**100**

The study found in examination of comorbid diseases of the patients that 14.4% of the patients have hypertension (HT) at the most, and it is followed by Diabetes Mellitus (DM) at the rate of 4%. 27.7% of the patients smoke. 52.2% of the patients were treated in COVID-19 service and 2.5% of the patients were treated in intensive care unit. Other patients received outpatient treatment. Only 2 of 326 patients died. Comorbid diseases, smoking habits, hospitalization and survival rates of the patients are given in Table 2.

**Table 2:** Comorbidity and Survival Condition of Patients

	Number (N)	Percentage (%)
**Comorbidities and smoking habit**		
HT	34	10.4
DM	13	4.0
CAD	7	2.1
COPD	19	5.8
CRF	2	0.6
Cancer	1	0.3
CVA	0	0.0
Pregnancy	1	0.3
Other Diseases	7	2.1
**Smoking**	90	27.7
**Hospitalization to Service**		
Present	169	52
Absent	156	48
**Intensive Care Unit Stay**		
Present	8	2.5
Absent	318	97.5
**Transfer**		
Present	0	0.0
Absent	326	100.0
**Survival Condition**		
Exitus	2	0.6
Right	324	99.4
**Total**	**326**	**100**

Pathological findings were found in 38.3% of the patients in examination of chest CT findings. The most common CT finding is ground glass opacity at the rate of 26.7% and it is followed by atelectasis at the rate of 12.3%. Bilateral involvement is present in all lesions of 20.2% of the patients. 7.7% of the patients have right lung involvement, and 5.8% have left lung involvement. Chest CT features of the patients are given in Table 3.

**Table 3:** Distribution of patients by CT findings

CT Findings	Number (N)	Percentage (%)
Normal	201	61.7
Bilateral Involvement	66	20.2
Unilateral Involvement	44	13.5
Right Involvement	25	7.7
Left Involvement	19	5.8
Ground Glass Opacity	87	26.7
Halo Sign	6	1.8
Air Bronchograms	15	4.6
Pleural Effusion	6	1.8
LAP	18	5.5
Septal Thickening	20	6.1
Atelectasis	40	12.3
Consolidation	23	7
Crazy Paving Pattern	8	2.5

The patients were split into 2 age groups as below and above 65 years old. These groups were compared to each other in terms of the length of intensive care unit stay, survival, and CT findings. The study found significant differences in the length of intensive care unit stay, exitus, normal CT, ground glass opacity, bilateral involvement, halo sign, pleural effusion, septal thickening, atelectasis, crazy paving pattern appearance between the group below 65 years old and the group above 65 years old. While the rate of intensive unit care stay and exitus is higher in the group above 65 years old (p<0.001, p=0.002 respectively), no significant difference was found in unilateral involvement, right involvement, left involvement, air bronchograms, nodule, LAP, pneumonic infiltration, and conslidation appearance between the two groups (Table 4).

**Table 4:** Comparison of the group below 65 years old and the group above 65 years old by intensive care unit stay, survival, and CT findings*

		<65 YEARS OLD		≥65 YEARS OLD		
		N	%	N	%	P
Intensive Care Unit Stay	Absent	270	84.9%	48	15.1%	0.000
	Present	1	12.5%	7	87.5%	
Exitus	Absent	270	83.6%	53	16.4%	0.002
	Present	0	0.0%	2	100.0%	
Normal	Absent	87	69.6%	38	30.4%	0.000
	Present	184	91.5%	17	8.5%	
Ground Glass	Absent	217	90.8%	22	9.2%	0.000
	Present	54	62.1%	33	37.9%	
Bilateral	Absent	232	89.2%	28	10.8%	0.000
	Present	39	59.1%	27	40.9%	
Unilateral	Absent	236	83.7%	46	16.3%	0.495
	Present	35	79.5%	9	20.5%	
Right	Absent	249	82.7%	52	17.3%	0.499
	Present	22	88.0%	3	12.0%	
Left	Absent	258	84.0%	49	16.0%	0.078
	Present	13	68.4%	6	31.6%	
Halo Sign	Absent	268	83.8%	52	16.3%	0.029
	Present	3	50.0%	3	50.0%	
Air Bronchograms	Absent	261	83.9%	50	16.1%	0.081
	Present	10	66.7%	5	33.3%	
Nodule	Absent	266	82.9%	55	17.1%	0.310
	Present	5	100.0%	0	0.0%	
Pleural Effusion	Absent	269	84.1%	51	15.9%	0.001
	Present	2	33.3%	4	66.7%	
Lap	Absent	259	84.1%	49	15.9%	0.055
	Present	12	66.7%	6	33.3%	
Septal Thickening	Absent	259	84.6%	47	15.4%	0.004
	Present	12	60.0%	8	40.0%	
Atelectasis	Absent	243	85.0%	43	15.0%	0.018
	Present	28	70.0%	12	30.0%	
Pneumonic Infiltration	Absent	266	82.9%	55	17.1%	0.310
	Present	5	100.0%	0	0.0%	
Consolidation	Absent	258	83.8%	50	16.2%	0.204
	Present	13	72.2%	5	27.8%	
Crazy Paving Pattern	Absent	268	84.3%	50	15.7%	0.000
	Present	3	37.5%	5	62.5%	

* Pearson Chi –Square Tests

In addition, patients hospitalized in the normal service (n= 169) and those hospitalized in the intensive care unit (n=8) were compared with each other in terms of age, comorbid factors and lung findings. A statistically significant difference was found between the groups in terms of age and COPD. COPD patients were significantly more hospitalized in the intensive care unit (p<0.05). As a result of the post-hoc analysis made to determine the difference between age groups; It was found that patients in the> 65 age group were hospitalized significantly more in the intensive care unit compared to the age group <18, 18–30, 31–50, and 51–64 years (p<0.01). Other parameters (other comorbid factors, pulmonary findings) did not have a significant effect on admission to the intensive care unit (p>0.05) (Table 5). Logistic regression analysis was performed to determine the effects of admission to the intensive care unit in patients with age and COPD. As a result of this analysis, age was determined as an independent predictor for intensive care unit admission (β = 0.091, P = 0.001). However, it has been found that COPD is not an independent predictor (β = 0.899, P = 0.321). Chest CT images of some of our patients are shown in figure 1.

**Table 5:** Comparison of Patients' Normal Service and Intensive Care Unit Hospitalization Status According to Age Groups, Comorbid Factors and Pulmonary Findings

	Normal Service Hospitalization n=169	Intensive Care Unit Hospitalization n=8	P Values
**Age**			
<18	2 (%66.7)	1 (%33.3)	.000*
19–30	48 (%100)	0 (%0)	
31–50	62 (%100)	0 (%0)	
51–64	26 (%100)	0 (%0)	
>65	31 (%81.6)	7 (%18.4)	
**Comorbidity**			
Hypertension	20 (%11.8)	1 (%12.5)	.955
DM	6 (%3.6)	1 (% 12.5)	.204
CAD	4 (%2.4)	1 (%12.5)	.091
COPD	8 (%4.7)	2 (%25)	**.015**
CRF	2 (%1.2)	0 (% 0)	.757
Malignancy	1 (%0.6)	0 (% 0)	.827
CVD	-	-	-
Pregnancy	1 (%0.6)	0 (% 0)	.827
Other	5 (%3.0)	1 (%12.5)	.145
Smoke	43 (%25.4)	0 (% 0)	.101
**According to Lung Findings**			
Normal	3 (%1.8)	0 (% 0)	.704
Bilateral Involvement	48 (%28.4)	3 (%37.5)	.579
Unilateral Involvement	23 (%13.6)	2 (%25)	.366
Right Lung Involvement	44 (%26.0)	3 (%37.5)	.473
Left Lung Involvement	41 (%24.3)	1 (%12.5)	.445

**Figure 1:** Chest CT findings of some patients in the study

**Figure:**
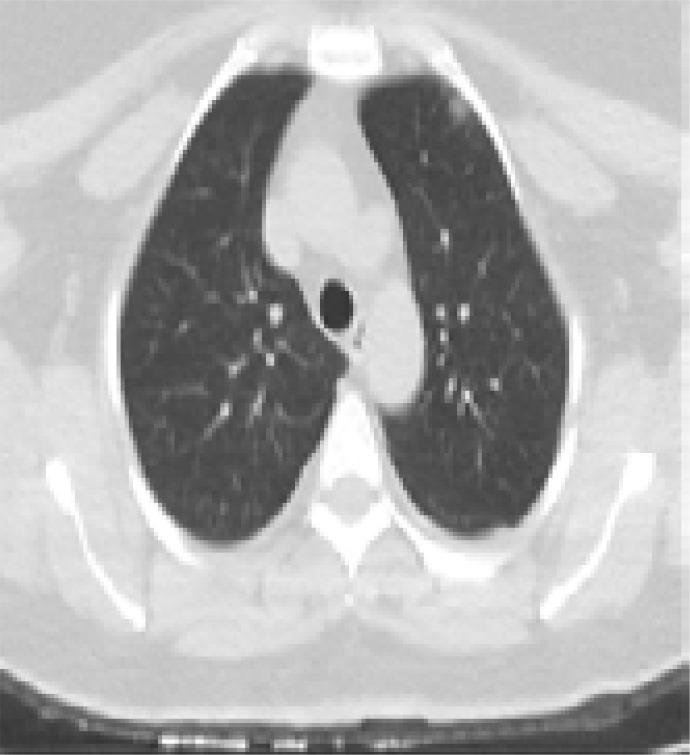
**A)** A 47-year-old male patient applied to hospital with complaints of fever, cough, and sore throat for 2 days. A nodule of ground glass density is found in anterior segment of upper lobe in left lung (Black arrow) in his tomography image. The patient was diagnosed with COVID-19 infection in early period.

**Figure:**
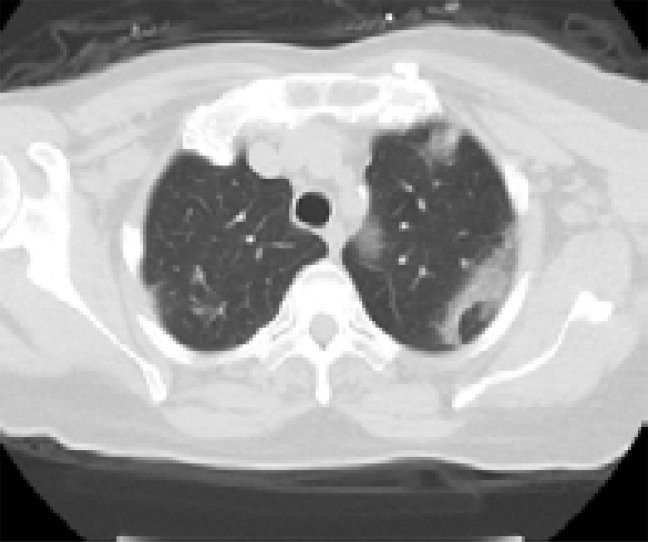
**B)** An 87-year-old patient with DM+HT+CAD history had the complaints of fever, shortness of breath, and cough for around 7–8 days. The patient applied to hospital due to increase in shortness of breath. Infiltration areas (black arrows) of peripheral ground glass density in all lobes of both lungs and small amount of effusion in both pleurae was found in the tomography picture of the patient.

**Figure:**
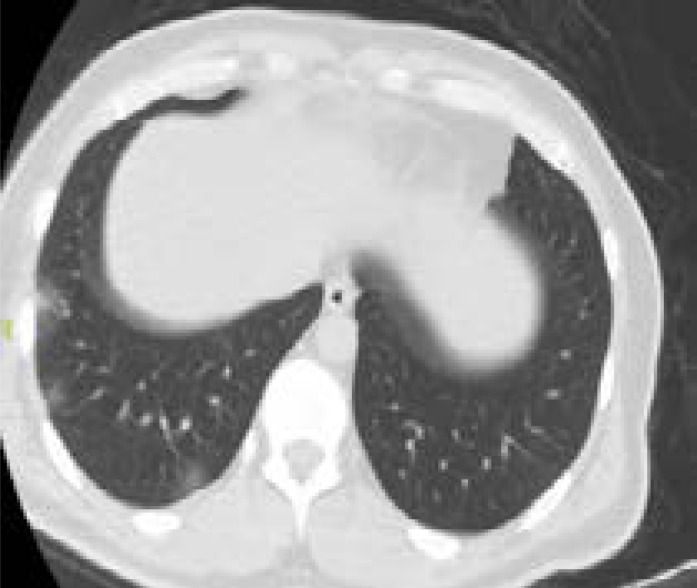
**C)** A 42-year old male patient applied to hospital with complaints of sore throat, fever, and cough for 3 days. Subpleural ground glass areas consistent with COVID-19 were found in basal segments of lower lobe of the right lung in the tomography picture of the patient. (black arrows)

**Figure:**
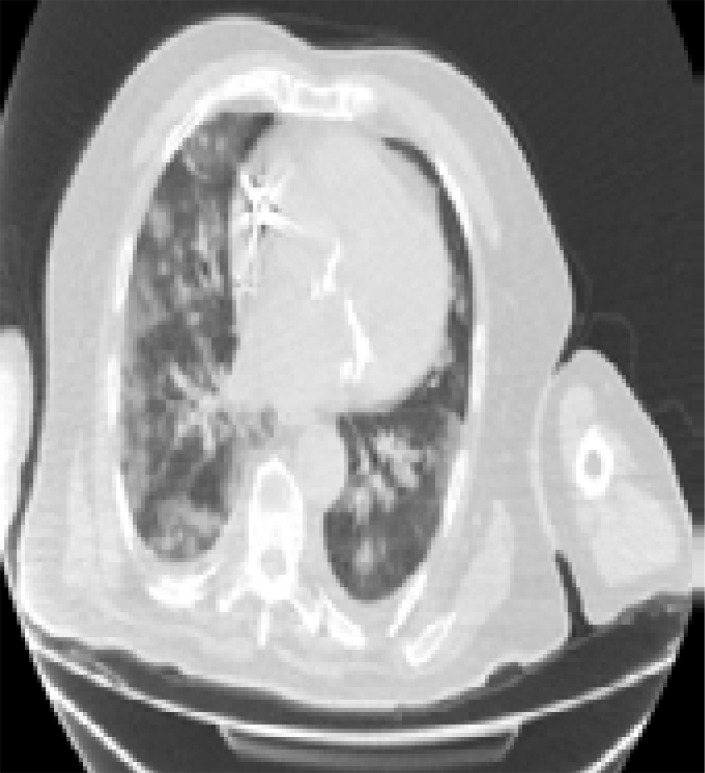
**D)** A 64-year-old female patient with HT history applied to hospital with complaints of arthralgia (joint pain), cough, and dyspnea for 5 days. Ground glass densities containing air bronchograms, more specifically in the left lung, were found in the tomography picture of the patient. (black arrows)

**Figure:**
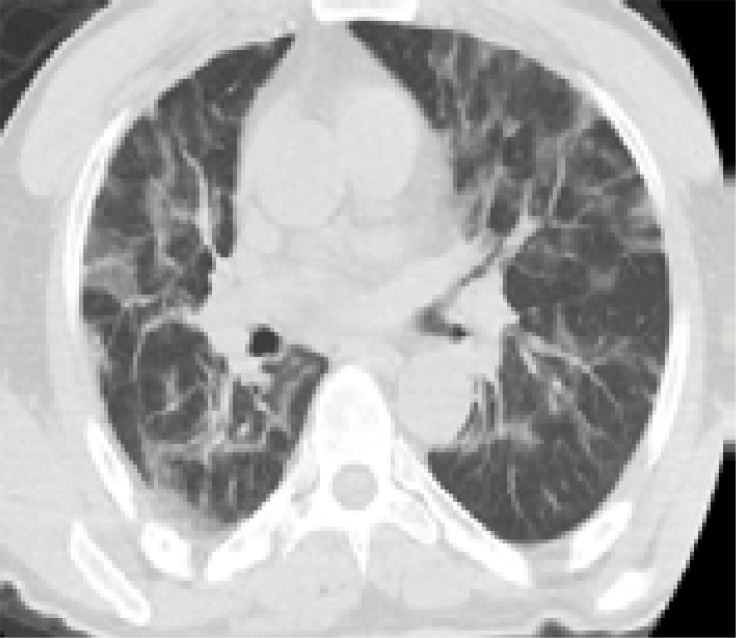
**E)** A 57-year old female patient applied to hospital with complaints of fatigue and cough for 2 weeks. Fibroatelectatic line in the lower lobe of right lung and nodular ground glass density (black arrows) were found in chest CT image of the patient.

**Figure:**
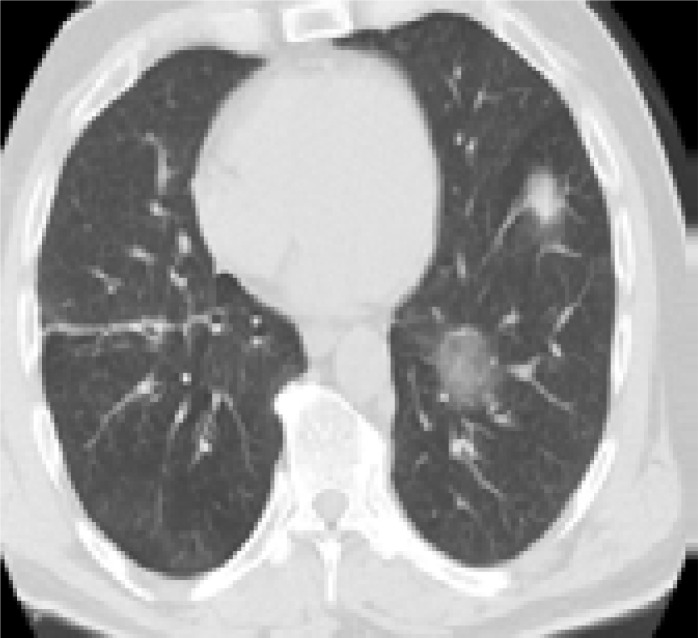
**F)** An 87-year old female patient with CAD+DM+HT history applied to hospital with complaints of cough and dyspnea for 1 week. Dispersed focal and reticular ground glass densities, more specifically in peripheral segments, in the parenchyma of both lungs were found in chest CT of the patient (black oval)

**Figure:**
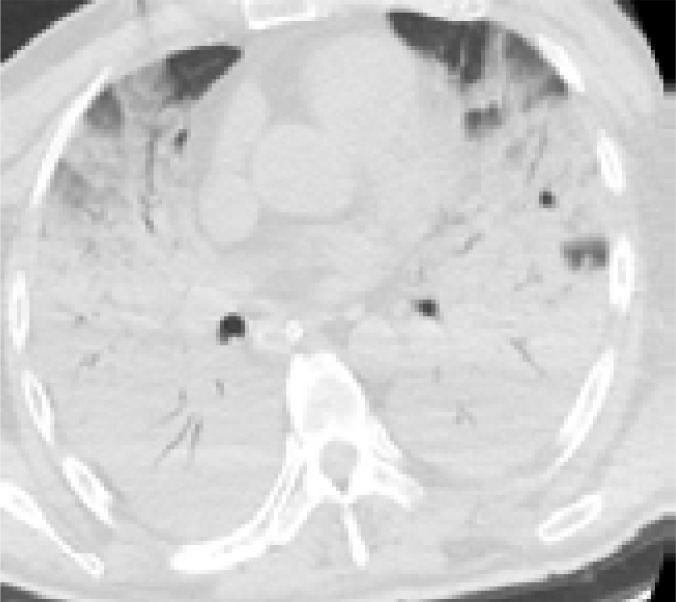
**G)** A 77-year old male patient with DM+HT history applied to hospital with complaints of cough, fever, and shortness of breath for 10 days. Dyspnea increased more specifically in the last 24 hours. Density increase in ground glass density, more specifically in the lower lobes, in both lungs, and interstitial and interseptal thickening, more specifically in partly peripheral segments, were found in chest CT image of the patient. (reticular ground glass areas /crazy paving pattern) (They are indicated in Black Oval shapes)

**Figure:**
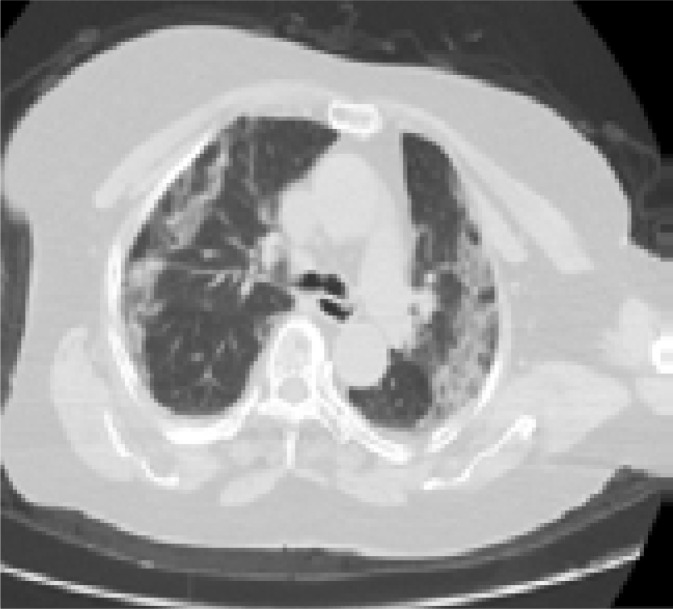
**H)** A 64-year old male patient with HT history had the complaints of fever, cough, and shortness of breath for 12 days. The patients applied due to progression of dyspnea for 2 days. Density increases of focal ground glass density, consolidations, and focal atelectasis appearances were found, more specifically in posterior segments and lower lobes of both lungs, suggesting a severe COVID-19 in chest CT image of the patient (They are indicated in black oval). The patient was intubated in our hospital and died in intensive care unit around 12 hours later.

**Figure:**
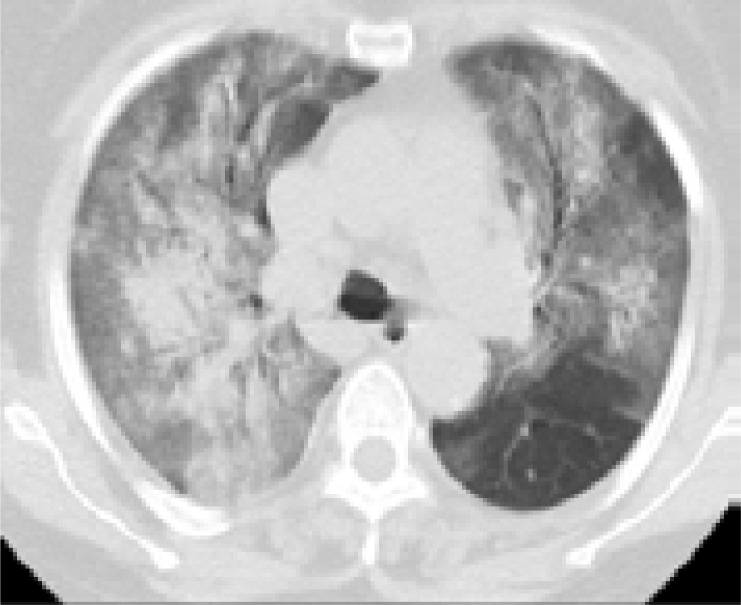
**I)** A 76-year old female patient with COPD history had the complaints of cough and joint pain for 10 days. No pathological finding was found in chest CT image of the patient which was scanned 7 days before application to the hospital. She applied due to excessive increase of dyspnea complaint. Opacities of peripheral subpleural parenchymal ground glass densities and consolidations were found in all lobes and segments of both lungs suggesting severe COVID-19 in chest CT image of the patient. (They are indicated in black oval shapes)

## Discussion

COVID-19 cases and the death rates due to this disease are rapidly increasing all over the world. Although the vaccine for COVID-19 has been produced and used today, there is no specific effective treatment for COVID-19 yet. Early diagnosis and treatment of the coronavirus disease are of vital importance. Even though RT-PCR is regarded as a gold standard for diagnosis of COVID-19, the false negative rates as reported in the range 10–40% may jeopardize efforts to curb the spread of the disease by early detection 9. The chest CT can be a more reliable, practical and faster alternative method for early diagnosis and treatment for COVID-19 patients^12^. The combined use of RT-PCR and chest CT is recommended in the diagnosis and treatment of patients presenting with COVID-19 clinic.^12^–^14^. We aim to set forth the general clinical features and chest CT findings of 326 COVID-19 RT-PCT (+) patients that applied to the hospital in detail.

A metaanalysis study by Li et al. on 10 studies including 1994 patients reported that the rate of male patients is higher than that of females in 9 out of these 10 studies. In addition, the most common symptoms in these studies are fever, cough, fatigue, myalgia, and dyspnea 20. A metaanalysis on 38 studies including 3062 COVID 19 patients in China reported that the most common symptoms are fever (80.4%), cough (63.1%), fatigue (46%), and myalgia (33%) 21. We found that 53.1% patients in our study are males, as well; this finding is consistent with the literature. Besides, the most common symptoms in the patients in this study are shortness of breath (21.8%) and cough (20.6%) and 43.3% of the patients are asymptomatic. We think that the difference between the findings of this study and the findings in the literature is related to the study period. During the study period, all potential cases were examined and treated in hospital in order to prevent the spread of the pandemic in line with the guidelines of Turkey Ministry of Health at the beginning of the pandemic 6. Therefore, too many asymptomatic cases applied to hospital. A metaanalysis by Yang et al. including 1576 infected patients reported that the most common comorbidities are HT (21.1%), DM (9.7%), cardiovascular disease (8.4%), and respiratory disease (1.5%) 21. Also, a study including 135 infected patients in China reported that 31.9% of the patients have an underlying disease and the patients most commonly have HT, DM, cardiovascular disease, and malignancy history 22. In this study, the patients have HT (10.4%), chronic obstructive pulmonary disease (5.8%), DM (4%), and coronary artery disease (2.1%) as comorbid disease; these findings are consistent with the literature. We think that the relatively less comorbid factors in the patients in this study are because of the plentitude of asymptomatic cases in the study.

A compilation published in July 2020 reported chest CT findings of COVID-19 along with their features. According to this study, ground glass opacity generally occurs in the early period of the disease and it is the most common chest CT finding. Consolidations occur in the late period (10–12 Days). However, they may also occur in the early period of severe pneumonia. Crazy-Paving pattern occurs in the progressive phase (5–9 days) of the disease or the early period of severe pneumonia. Fibrosis occurs in the late period of the disease and the severe pneumonia. Halo sign defines the nodule or mass surrounded by ground glass areas and it is encountered infrequently. Pleural thickening is seen as the finding of late period 24.

A study by Ai et al. on 1014 cases in China found that 59% of the patients are RT-PCR (+) and 88% have chest CT (+). It indicates that chest CT is specific to COVID-19 diagnosis at the rate of 97% based on RTPCR positive result of the patients. In addition, consolidation is found in 50% of the patients; ground glass opacity is found in 46% of the patients in examination of chest CT findings of the patients and findings of 90% of the patients are bilateral.^11^ Also, a study by Chung et al. that examines chest CT images of 21 patients reported that 57% of the patients have ground glass opacity and 29% have both ground glass areas and consolidation. Besides, the study found bilateral lung involvement in 76% of the patients. Also, 33% of these patients have peripheral dispersion and 19% have crazy-paving pattern 12. A study by Pan et al. which examines chest CT images of 61 COVID-19 patients reported that 22.2% of the patients have ground glass areas, 85.7% have patchy/ punctate ground glass opacities 14. Chest CT images of 61.7% of the patients in this study are considered as normal. Ground glass opacities were found in 26.7% of the patients and 12.3% of the patients have atelectasis and 7% of the patients have consolidation. Crazy paving pattern was found in 2.5% of the patients; halo sign finding was found in 1.8% of the patients. 20.2% of the patients have bilateral lesions. We think that the reason for relatively infrequent existence of CT findings in this study is the fact that the number of asymptomatic cases is higher than the number of normal CT.

Many previous studies reported that age is a significant factor for mortality, length of hospital stay, and viral clearance in COVID-19 25–27. It was reported in an analysis of 44.672 cases that were diagnosed in China as of February 11, 2020 that the mortality rate was 2.3% in general. However, the mortality rate is 8% in patients between the ages of 70–79 and 14.8% in patients between the ages of ≥80 28. This study found a significant difference in intensive care unit stay, exitus, normal BT, ground glass, bilateral involvement, halo sign, pleural effusion, septal thickening, atelectasis, and crazy paving pattern appearance between the group below 65 years old and the group of 65 years old and above. While the rates of intensive care unit stay [%87.5 vs %12.5 p<0.001] and exitus [ %100 vs %0 p=0.002] are higher in the group above 65 years old, bilateral involvement is found more frequently in the group below 65 years old. In addition, age has been shown to be an independent predictor of intensive care unit admissions.

In a review published in 2020, it is reported that comorbid conditions such as DM, Hypertension, Coronary Artery Disease (CAD), Chronic Lung Diseases increase the clinical severity and worsen the prognosis of COVID-19 22. In our study, similarly with the literature, the rates of admission to the intensive care unit were found to be significantly higher in patients with COPD comorbidity. However, unlike the literature, we found that other comorbid factors did not significantly affect admissions to intensive care. We think that the reason for this difference is the low number of patients hospitalized in the intensive care unit in our study.

## Limitations

COVID-19 patient management strategies change constantly in the light of scientific data since the beginning of the pandemic. This study includes the cases between March 11, 2020 when the first case was reported in Turkey and June 30, 2020. There are many changes in patients that apply to hospital and the profile of hospitalized patients depending on many changes in diagnosis and treatment strategies for COVID-19 after June 30, 2020 and the course of the pandemic. Therefore, new studies that include the patients after June 30, 2020 are needed. We plan to extend our findings in this study with additional analysis including these periods.

## Conclusion

In examination of chest CT findings of the patients, the most normal CT findings were found during the early periods of the pandemic depending on the COVID-19 case management strategy. The most frequent pathological chest CT finding is ground glass opacity. The rates of intensive care unit stay and mortality are significantly higher in the patients above 65 years old compared to the patients below 65 years old.

## Declarations

### Availability of data and material

Yes
